# Cracking and Fiber Debonding Identification of Concrete Deep Beams Reinforced with C-FRP Ropes against Shear Using a Real-Time Monitoring System

**DOI:** 10.3390/polym15030473

**Published:** 2023-01-17

**Authors:** Nikos A. Papadopoulos, Maria C. Naoum, George M. Sapidis, Constantin E. Chalioris

**Affiliations:** Laboratory of Reinforced Concrete and Seismic Design of Structures, Civil Engineering Department, School of Engineering, Democritus University of Thrace, 67100 Xanthi, Greece

**Keywords:** reinforced concrete (RC), deep beams, carbon-fiber-reinforced polymer (C-FRP) rope, shear, retrofitting, piezoelectric lead zirconate titanate (PZT), structural health monitoring (SHM), debonding, damage detection

## Abstract

Traditional methods for estimating structural deterioration are generally costly and inefficient. Recent studies have demonstrated that implementing a network of piezoelectric transducers mounted to critical regions of concrete structural members substantially increases the efficacy of the structural health monitoring (SHM) method. This study uses a recently developed electro-mechanical-admittance (EMA)-based SHM system for real-time damage diagnosis of carbon FRP (C-FRP) ropes installed as shear composite reinforcement in RC deep beams. The applied SHM technique uses the frequency response measurements of a network of piezoelectric lead zirconate titanate (PZT) patches. The proposed strengthening methods using C-FRP ropes as ETS and NSM shear reinforcement and the applied anchorage techniques significantly enhanced the strength and the overall performance of the examined beams. The retrofitted beams exhibited increased shear capacity and improved post-peak response with substantial ductility compared with the brittle failure of the non-strengthened specimens. The health condition and the potential debonding failure of the applied composite fiber material were also examined and quantified using the proposed SHM technique. Damage quantification of C-FRP ropes is achieved by comparing and assessing the values of several statistical damage indices. The experimental results demonstrated that the proposed monitoring system successfully diagnosed the region where the damage occurred by providing early warning of the forthcoming critical shear cracking of concrete and C-FRP rope debonding failures. Furthermore, the internal PZT transducers showed sound indications of the C-FRP rope’s health condition, demonstrating a direct correlation with the mechanical performance of the fibers.

## 1. Introduction

The classification of reinforced concrete (RC) beams is conventionally differentiated into typical or slender and deep ones, respectively, according to their shear span to depth (α/d) ratio. A lower to 2.5 ratio usually leads to shear-critical behavior with considerable transversal stiffness, while an increased one of more than 2.5 is mainly characterized by flexural responses [1,2]. The failure mechanism of shear-critical RC beams develops brittle behavior and abrupt loss of bearing ability leading to sudden collapse, which could hinder the economy and cause severe human losses. In addition, concrete’s compressive strength, reinforcement arrangement, and static model also affect beams’ structural performance.

The behavior of the deep beams is considered differently in Eurocode 2 (EN 1992-1-1:2004), proposing an alternative design and analysis process, such as the truss method followed by modeling the beam with strut and tie elements for the transmission of the flexural and tensional stresses to the support points. Deep beams are often met in the stairwell multi-story RC structures and the coupling beams in shear walls. Still, their high depth also usually allows ample space for designing openings to support functional facilities’ installation [3]. Experimental studies have also been conducted on RC beams with openings investigating the effect of their size and shape, and the mechanisms of crack initiation and propagation on the behavior of the beam [4,5,6].

In the last decades, externally epoxy-bonded fiber-reinforced polymers (FRP) have been widely used for rehabilitating, strengthening, retrofitting, and repairing RC structures such as shear strengthening beams [7], torsional strengthening beams [8,9], and shear strengthening beam-column joints [10,11], thanks to their merits of high tensile and fatigue strength, their light weight, and their excellent resistance to corrosion [12]. In particular, carbon-fiber-reinforced polymers (CFRPs) present higher performance in the abovementioned properties than the others FRPs. In addition, CFRP has high stiffness, which can also meet the reinforcement needs of concrete structures when considering the deformation of components [13,14]. On the other hand, there are scruples concerning the implementation of FRP materials in real structural applications regarding premature debonding failure and low fire resistance durability [6,15].

Premature debonding reduces the retrofitting efficiency of the FRPs in RC structures resulting in a lower actual strain response compared to the total potential designed capacity due to the lack of coherence in the interfacial zone between the FRP material and concrete surface. In addition, many existing studies in the broader literature have reported that the ordinarily recognized failure mode of strengthened RC members with externally epoxy-bonded FRP composites is premature debonding or delamination of the FRP layer from the concrete substrate. The latter is caused by the high concentration of stresses and accompanied by brittle catastrophic failure [6,16]. Especially in T-shaped RC structural members and U-shaped strengthening scheme applications, the early debonding failure of the externally applied FRP is caused due to the existence of the slab, which provides limited applicability for wrapping the FRP retrofitting materials around the cross-section or/and accessibility to apply the end anchorage properly.

A couple of promising techniques have emerged to prevent early debonding of FRP under low strains. Primarily, a proposed method known as near surface mounted (NSM) refers to the application of FRP plates and rods which were inserted and bonded with epoxy adhesive in pre-drilled grooves formed to the mass of the concrete. It was observed that the grooves’ depth affects the technique’s effectiveness but constitutes an agent of limitation when the requisite thickness of the concrete cover is insufficient [17,18,19,20]. Furthermore, more alternative methods have been proposed in the literature for shear strengthening of RC beams: (a) steel or carbon FRP (C-FRP) bars embedded through section (ETS) reinforcement [21,22,23], (b) FRP ropes have been examined as a shear retrofitting reinforcement in multiple applications, as confining reinforcement in RC columns [24,25] as a shear retrofitting reinforcement in RC beams [26], as an anchorage reinforcement in FRP shear-strengthened RC beams [27,28].

Moreover, Chalioris et al. [29] investigated the effectiveness of a new retrofitting technique to improve the capacity and the performance of shear-critical RC deep beams without stirrups using C-FRP ropes as the only transverse reinforcement. Two techniques to install the C-FRP ropes were examined: (1) in the beam with rectangular cross-section, one vertical and one shear-favorably inclined single-link rope that has been internally applied through the web as ETS reinforcement and (2) in the flanged beam with a T-shaped cross-section, two vertical U-shaped double-link ropes are applied at the web’s perimeter as NSM reinforcement. Additionally, the effectiveness of the anchorage of the C-FRP ropes has also been examined in both cases. In conclusion, the experimental performance of the applied shear-strengthening methods showed promising results as the examined shear-critical beams finally led to flexural failure. The findings of the experimental study were successfully published [29].

Despite the advantages of the proposed techniques, significant drawbacks still need further investigation. Specifically, in the field of strengthening RC structures with FRP composite materials, some previous studies were dedicated to identifying the damage of epoxy-bonded FRP reinforcement at early damage states, proposing non-destructive testing (NDT) methodologies. However, most do not apply to large and complex structures due to structural limitations and the need for prior knowledge of the damage location. Furthermore, most NDT methods are time-consuming, expensive, require access in situ, and cannot apply continuous and real-time monitoring. Infrared thermography [30,31], ultrasonic C-scan detection, acoustic emission (AE), and resistance strain gauges [32] are commonly used to check the integrity of composite materials [33,34,35]. The latter is predominantly destined for static measurements and achieves low transverse sensitivity [36].

On the other hand, a handy tool for evaluating the performance of the proposed retrofitting techniques could be the implementation of the structural health monitoring (SHM) method by measuring structural responses in real-time and identifying abnormalities or/and damage at early stages [2]. SHM methods can generally be categorized into two types concerning the sensing methodology: active and passive.

Various experimental and analytical studies have pointed out the emerging and efficient electro-mechanical impedance (EMI) method in SHM applications in RC elements [37,38] and sub-assemblages’ structures [39,40,41]. This method is based on the application of smart piezoelectric materials especially lead zirconate titanate (PZT) transducers applied for feasible continuous monitoring [42,43,44] of FRP external strengthening members [45]. Thanks to the merits of the PZTs, which can act as actuators and sensors simultaneously, the measurements of their electro-mechanical signatures can provide indications of internal damage detection [46,47]. Moreover, it might be easily implemented in a non-expensive way for continuous and real-time monitoring of complex infrastructures and non-easily accessible or non-accessible members within a wireless system [48,49].

Recent research has indicated that implementing a grid of PZT patches in areas of potential damage development essentially increases the efficacy and precision of SHM methods for damage detection, providing reliable structural monitoring in RC elements [50,51]. Moreover, recent studies have emerged on the advantages of the piezo materials versus the application of strain gauges investigated under the same conditions exhibiting a much higher signal-to-noise ratio and transverse sensitivity [36].

Although it is widely accepted, the application of strain gauges suffers from some limitations due to the a priori assumption of the uniform strain field, which practically recanted when cracking occurred [52]. In addition, strain gauges are locally limited, while PZT transducers can act in a wide spatial region. However, a combination of both methods could exhibit supplementary data for verifying the attained data [53].

Several studies have examined SHM techniques applied to concrete. Still, very few have investigated the monitoring of fiber-reinforced concrete with dispersed synthetic macro-fibers and C-FRP-strengthened structural members [54,55].

This study’s experimental part includes applying a novel monitoring system of two shear-strengthened beams subjected to four-point bending loading for damage detection and assessment. The proposed SHM method is an EMI-based PZT-enable wireless impedance/admittance monitoring system (WiAMS). The voltage frequency response of the PZT patches installed in different locations of the specimen has been measured at varying levels of the applied loading and corresponding structural health conditions (damage states). A quantitative assessment of the examined damage levels using values of the statistical damage index is also attempted.

The objective of this study is to address a new monitoring experimental method using WiAMS in C-FRP ropes to diagnose damage in shear-critical RC beams retrofitted with composite materials to predict the forthcoming brittle failure at early damage stages, such as at the onset of diagonal cracking or premature C-FRP debonding. The damage indices, namely the root mean square deviation (RMSD) and “moving RMSD” (mRMSD), are used for the localization process. Damage quantification using conventional static metrics such as RMSD are sometimes found to be ineffective in identifying the location of the damage. For this reason, the present work uses the dynamic metric mRMSD as an alternative [56].

The efficiency of the novel retrofitting technique against the shear of RC deep beams without stirrups using C-FRP ropes as the only shear reinforcement is also evaluated herein using the measurements of the adopted SHM method. The developed method is implemented to evaluate both ETS and NSM retrofitting systems and to investigate the ability of the PZT-enabled SHM system to detect the changes to the retrofitted region and, specifically, the identification of the debonding of the installed C-FRP ropes. The experimental measurements have been conducted using custom-made wireless monitoring devices (called WiAMS) and the implementation of PZT patches mounted to the ropes, the steel rebars, and the concrete surface of the tested specimens [57]. Furthermore, determining the sensitivity of the developed PZT-enabled EMI-based method in damage detection as far as the formed cracks’ location, distance, direction, and width is also an aspect of this experimental research.

## 2. Experimental Program

### 2.1. Characteristics of the Deep Beams

**Figure 1:**
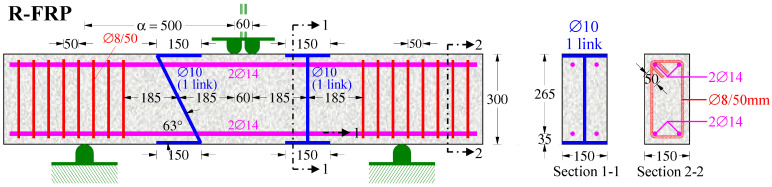
Geometry and reinforcement details of the beam R-FRP.

**Figure 2:**
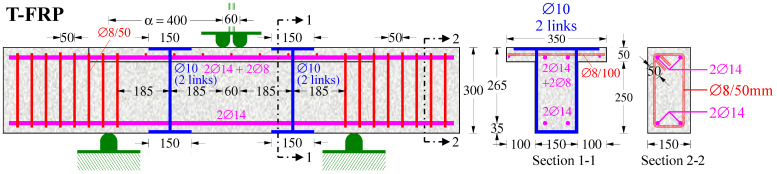
Geometry and reinforcement details of the beam T-FRP.

The test project of this paper includes two RC deep beams with length L = 1.6 m under a monotonic four-point load. The first beam was formed in a rectangular cross-section with an abbreviated code name “R-FRP”, with width b = 150 mm, height h = 300 mm, effective depth d = 265 mm, and shear span α = 500 mm (α/d = 1.89), as illustrated in Figure 1. The second beam was formed in a T-shaped cross-section with the coded name “T-FRP”, with flange width b_f_ = 350 mm, web width b_w_ = 150 mm, height h = 300 mm, effective depth d = 265 mm, and shear span α = 400 mm (α/d = 1.51), as shown in Figure 2.

Both of the specimens consisted of the same tensional and compressional longitudinal steel rebars of 14 mm (∅14) diameter. Two of the rebars were located on the top and the other two were the bottom bars. C-FRP ropes of Sika company (SikaWrap FX-50C) were used, and two different shear strengthening techniques were examined. The first technique was internally applied to the beam with a rectangular cross-section “R-FRP”, implementing the ropes invasively through the middle of the beam’s web. At the right shear span, one single-link rope was vertically installed, and a second one with around 52 degrees of inclination at the left was installed, respectively. Both were installed as ETS reinforcement.

Regarding the shear strengthening technique for the flanged beam “T-FRP”, two double-link C-FRP ropes were vertically placed in epoxied U-shaped notches that had been grooved at the perimeter of the web at each shear span as NSM reinforcement.

The process of the implementation of both techniques can be summarized in the below steps for ETS and NSM techniques:

*ETS installation procedure of the C-FRP rope in beam “R-FRP”*

1. Drilling of the holes for the inserted ropes and forming the tassels for the anchorage’s adoption;
2. Removing the trapped dust inside the drilled holes with compressed air and a specific tool brush;
3. Impregnation of the rope to epoxy resin according to the technical data sheet of the manufacturer
4. Filling the grooves of the anchorage’s tassel with epoxy resin;
5. Insertion to the holes of the impregnated C-FRP (PZT-bonded) ropes;
6. Filling of the holes with epoxy resin to eliminate voiding and to enhance coherence among the inserted materials;
7. Final forming of the anchorages by subjecting tension to the end of the ropes;
8. Adding extra epoxy resin to smooth the grooves up to the beams’ surface.

*NSM installation procedure of the C-FRP rope in beam “T-FRP”*

1. Engraving and carving of channels for the ropes’ insertion and formation of the tassels for the anchorage’s adoption;
2. Removing the dust inside the carved channels with compressed air;
3. Impregnation of the rope to epoxy resin according to the technical data sheet of the manufacturer;
4. Insertion and gliding of the impregnated C-FRP ropes into the formed channels;
5. Filling the grooves of the anchorage’s tassel with epoxy resin;
6. Filling the channel with epoxy resin to enhance coherence among the inserted materials.

### 2.2. Materials

The C-FRP rope used consisted of a bunch of unidirectional and flexible carbon fibers. According to the manufacturer, the nominal cross-section area was 28 mm^2^, the minimum elongation at break was 1.6%, and the modulus of elasticity of the carbon fibers before impregnation was 240 GPa. The C-FRP rope was soaked in epoxy resin (Sikadur-52) with the described in Table 1 properties. In addition, the properties of the impregnated C-FRP rope and the epoxy paste (Sikadur-330) used to fill and seal the drilled grooves are also presented in Table 1.

Compression and splitting tests of standard concrete cylinders (150/300 mm) were also performed, and the mean compressive and tensile strengths of the concrete of the beams on the day of testing were 28.0 MPa and 2.70 MPa, respectively. Furthermore, the maximum aggregate coarse size of the casted mixed concrete was 16 mm.

Additionally, the yield tensile strength of the ∅14 deformed steel rebars was 580 MPa, while the corresponding strength of the ∅8 mild steel stirrups was 310 MPa.

### 2.3. Test Setup

**Figure 3:**
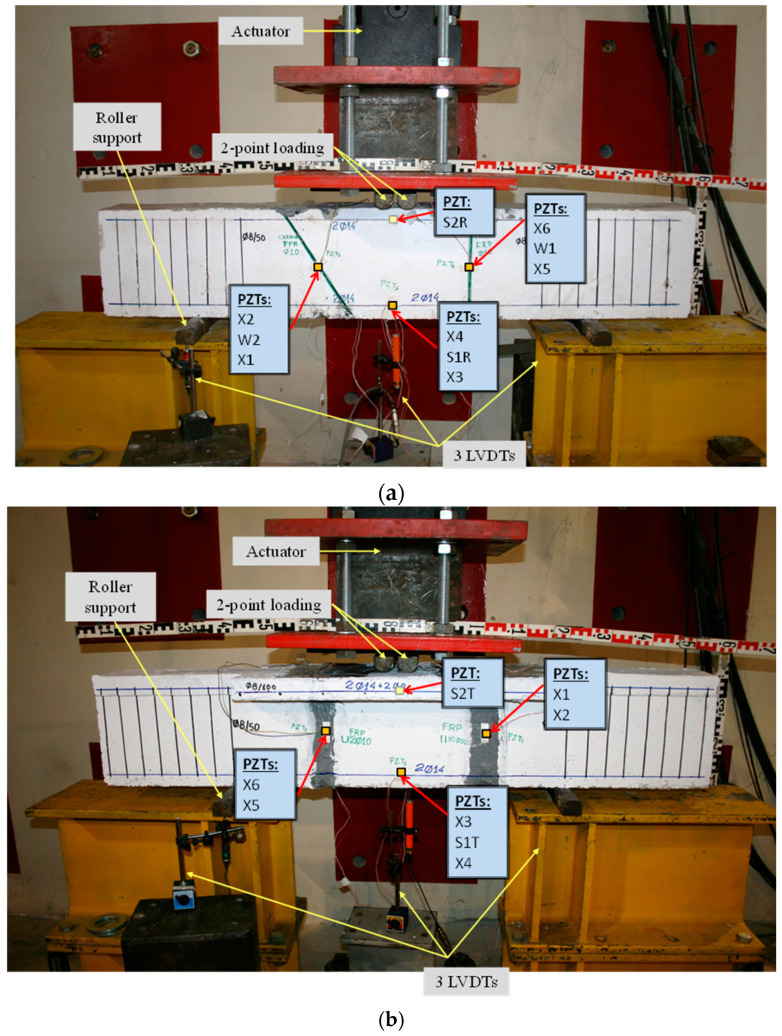
Test setup and instrumentation. (**a**) R-FRP deep beam with a rectangular cross-section and (**b**) T-FRP deep beam with a T-shaped cross-section.

Both examined beams were subjected to a typical four-point bending experimental testing setup, as illustrated in Figure 3.

A hydraulic piston operated by a servo-controlled machine subjected the beams’ surface to a monotonic forcing through two hardened steel rollers evenly spaced 60 mm apart. The tested beams were edge-supported using two steel roller supports on either side. A load cell was used to record the consistent increment of the applied load with 0.05 kN accuracy, and three linear variable differential transducers (LVDTs), two at the supports and one at the mid-span, were used to measure the deflections of the beams with 0.01 mm and 0.005 mm accuracy [29].

### 2.4. Electro-Mechanical Impedance (EMI) Method

The EMI method employs the application of PZT transducers and its principal characteristic feature, which is based on the advantages of the piezoelectric phenomenon. A superficial electric charge is generated under the subjection of mechanical stress, and vice versa; mechanical vibration is produced by applying an electric field. Thus, by exploiting the piezoelectric phenomenon via the actuation and the developed vibrations of bonded or embedded PZT transducers to a host structure, any alteration to its mechanical impedance (or the inverse admittance) is also reflected in the changes to the extracted electrical signal of the PZT (voltage response or frequency response). The interaction between PZT and the RC element is captured in the form of an admittance signature consisting of the conductance (real part) and the susceptance (imaginary part). As a result of these interactions, structural characteristics are reflected in the signature as expressed in the following Equation (1) for complex admittance, $\bar{Y}$, of the mounted PZT patch:

(1) $$\bar{Y}=\frac{\bar{I}}{\bar{V}}=G+Bj=4\omega j\frac{L^{2}}{h}\left[\bar{\varepsilon_{33}^{T}}-\frac{2d_{31}^{2}\bar{Y^{E}}}{\left(1-\nu \right)}+\frac{2d_{31}^{2}\bar{Y^{E}}}{\left(1-v\right)}\left(\frac{Z_{a,eff}}{Z_{s,eff}+Z_{a,eff}}\right)\left(\frac{\tan kL}{kL}\right)\right]$$

where $\bar{V}$ is the harmonic alternating voltage supplied to the circuit, $\bar{I}$ is the current passing through PZT, *G* is the conductance (the real part of admittance), *B* is the susceptance (the imaginary part of admittance), *j* is the imaginary unit, *v* is the angular frequency, *L* is the half-length of the patch, *h* is the thickness of the patch, $d_{31}$ is the piezoelectric strain coefficient of the PZT, $Z_{a,eff}$ is the short-circuited effective mechanical impedance, $Z_{s,eff}$ is the effective structural impedance, *v* is Poisson’s ratio, *k* is the wave number related to the angular frequency, $\bar{Y^{E}}$ is the complex Young’s modulus of elasticity under constant electric field, and $\bar{\varepsilon_{33}^{T}}$ is the complex electric permittivity of PZT patch along axis “3” at constant stress.

Any damage to the RC beam that changes its mass and stiffness characteristics will cause the structural parameters to change and will thus alter the effective structural impedance, which in turn changes the admittance as defined by Equation (1), thus serving as an indicator of the state of health of the element [49].

**Figure 4:**
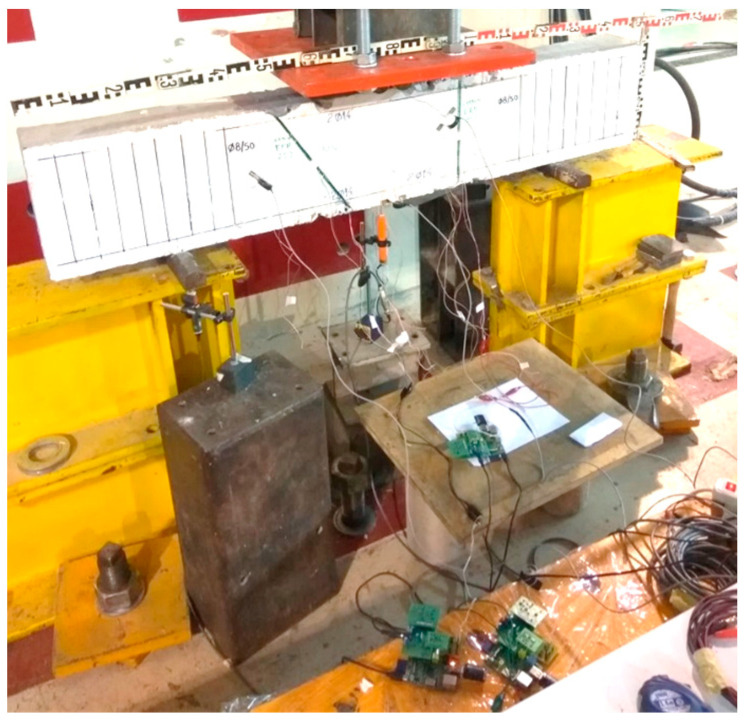
WiAMS devices of the proposed SHM method during the RC deep beam testing.

In this experimental work, the adopted method for detecting any potential damage developed to the concrete mass or the installed C-FRP ropes comprises the employment of PZT transducers excited with the amplification of a sinusoidal harmonic voltage of 2.5 V in terms of a specific frequency range between 10 to 250 kHz per step of 1 kHz. The PZT transducer operates as an actuator and a sensor concurrently. The subjected voltage signal and the records of the measurements of the extracted electro-mechanical signatures were implemented through a developed wireless device with the acronym WiAMS [46,49,58,59]. This custom-made device can also perform a vast scale of calculations in a short time, having high processing power and being held remotely (Figure 4).

**Figure 5:**
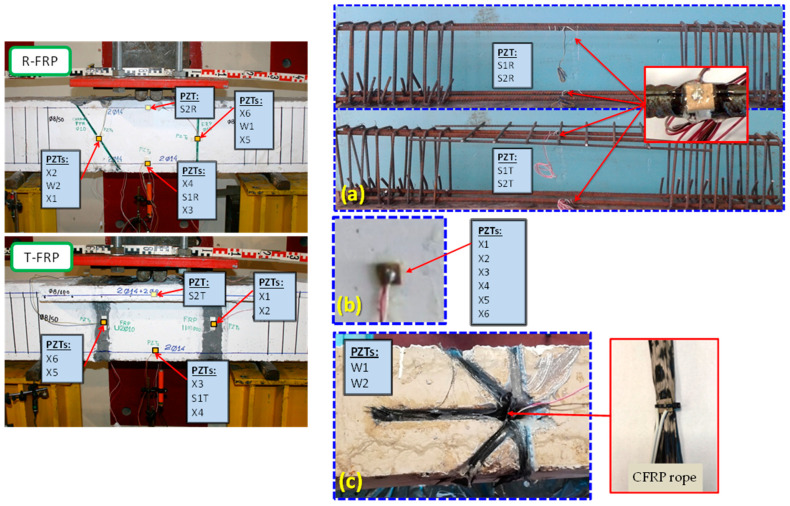
Installation of PZTs transducers: (**a**) epoxy-bonded PZTs on the reinforcing steel bars, (**b**) externally epoxy-bonded PZTs mounted to the concrete surface, and (**c**) embedded PZTs transducers on C-FRP ropes.

**Figure 6:**
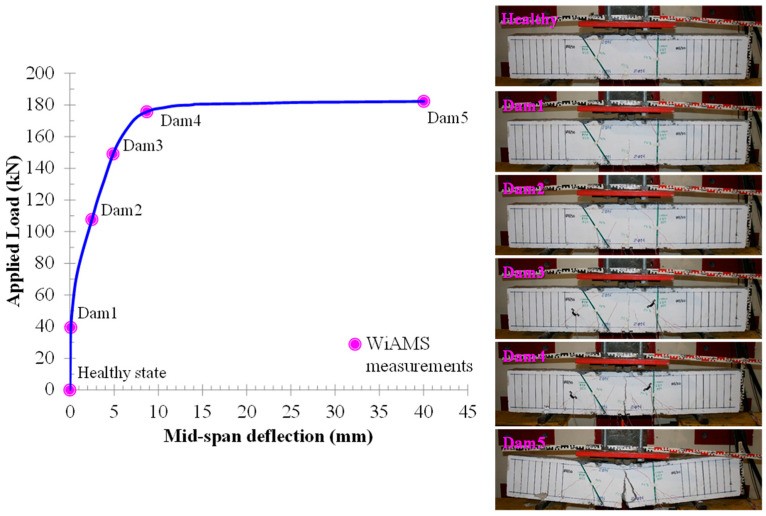
Cracking pattern and experimental behavior of specimen R-FRP.

The initial EMI measurements were carried out to the beam’s pristine condition, declared healthy, and used as a baseline state. Thence, the measurements were repeated at different potential damage states, the results were compared, and the structural integrity was evaluated through statistical indices (Figure 5 and Figure 6).

### 2.5. Installation of PZT Transducers

For both beams, the same small and thin type of PZT transducers were installed with the dimensions 10 × 10 × 2 mm. Their sign mark was PIC151, manufactured by the PI Ceramics company. The PZT patches were placed on the beams as described below:

a. Two epoxy-bonded PZTs for each beam were located, the first one on the surface of the one steel tensional reinforcing bar and the second one on the surface of the steel flexural reinforcing bar after proper flattening of the bar (internal PZTs), both located in the middle of the steel bar and named as S1 and S2, respectively. An extra layer of the epoxy adhesive was also used thoroughly on the top of each PZT waterproofing and it protects the patches during the concrete casting and curing process to be devoid of noise effects in their extracted electro-mechanical signatures (Figure 5a).
b. Externally epoxy-bonded PZTs mounted to the concrete surface of the beams (external PZTs). A high shear modulus epoxy adhesive with a small thickness was applied to bond the PZTs. Four PZT patches were surficially bonded on the mid-height dimension on the left and right shear span on both sides near and along the length of the ropes’ position. Two more PZTs were externally placed on both sides in the bottom mid-span of the beams. The exact position of the PZT patches is illustrated in Figure 5b. The PZTs’ codified names are denoted as “X1”, “X2”, “X3”, “X4”, “X5”, and “X6”.
c. Two epoxy-bonded PZTs were also internally placed on the R-FRP beam. The PZT patches were simultaneously embedded with the insertion of the ropes inside each drilled hole. The PZT patch was embodied with the rope using an epoxy adhesive and tying the welded wires with the rope carefully using a tire-up component and a steel wire to achieve the final designed position of the PZT patch inside the hole. The targeted tying position was selected so that the PZT patch finally ended up bonded internally to the drilled hole in the middle of the traversing length of the rope. The steel wire was used as an assistant tool for driving the rope, the PZT patch, and its wires passing through the hole and then removed. The two patches are denoted as W1 and W2, respectively (Figure 5c).

## 3. Results and Discussion

### 3.1. Loading Test

**Figure 7:**
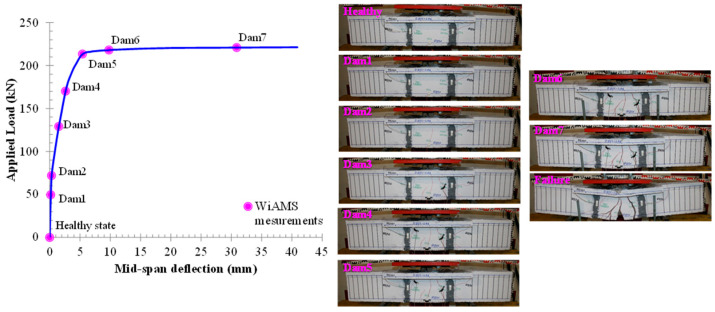
Cracking pattern and experimental behavior of specimen T-FRP.

The overall behavior of the beam specimens with rectangular and T-shaped cross-sections, in terms of applied load versus mid-span deflection curves, is demonstrated in Figure 6 and Figure 7, respectively. An extended description of the overall behavior of the beams can be found in the previous work of Chalioris et al. [29]. The cracking pattern of the failure of the tested beams is also illustrated in Figure 6 and Figure 7.

### 3.2. Damage Quantification

The scope of the damage quantification process should not be limited only to proving the damage’s existence but also to estimating its qualities, such as the location, width, and depth of the crack/cracks. Furthermore, the process should be orientated to the instrumented evaluation and quantification of the severity of the damage. The extracted data are analyzed and evaluated in this scope through statistical indices. The elaboration of the electro-mechanical signatures through statistical analysis could be a valuable tool in converting the variations of the EMI signatures between the healthy condition and any subsequent ones to damage index metrics.

Plenty of proposed indices could be met to the extent of the literature, with the most commonly used being those below:

1. RMSD: root mean square deviation;
2. MAPD: mean absolute percentage deviation;
3. CC: coefficient of correlation.

In this study, the commonly used damage indices root mean square deviation RMSD and MAPD were primarily applied for the statistical analysis of the EMI signatures. The expression of the traditional RMSD and MAPD indices are also presented below in Equations (2) and (3), respectively:

(2) $$\mathrm{RMSD}=\sqrt{\frac{\sum_{r=1}^{M}{\left({\left|V_{p}\left(f_{r}\right)\right|}_{D}-{\left|V_{p}\left(f_{r}\right)\right|}_{0}\right)}^{2}}{\sum_{r=1}^{M}{\left({\left|V_{p}\left(f_{r}\right)\right|}_{0}\right)}^{2}}}$$

(3) $$\mathrm{MAPD}=\frac{1}{M}\overset{M}{\underset{r=1}{\sum }}\frac{|\left|V_{p}{\left(f_{r}\right)}_{D}-V_{p}{\left(f_{r}\right)}_{0}\right||}{V_{p}{\left(f_{r}\right)}_{0}}$$

Furthermore, the promising moving RMSD (mRMSD) index was also applied as an additional tool for enhancing better evaluation levels. In Equation (4), the expression of the mRMSD index is described.

(4) $${\mathrm{mRMSD}}_{a=1,\;b-n}=\sqrt{\frac{\sum_{a=1}^{n}{\left({\left|V_{p}\left(f_{r}\right)\right|}_{D}-{\left|V_{p}\left(f_{r}\right)\right|}_{0}\right)}^{2}}{\sum_{a=1}^{n}{\left({\left|V_{p}\left(f_{r}\right)\right|}_{0}\right)}^{2}}}$$

Additionally, the recently proposed approach of the moving MAPD (mMAPD) index was implemented to insert more tools in the damage quantification process. The expression of the mMAPD index is also described in Equation (5).

(5) $${\mathrm{mMAPD}}_{a=1,\;b-n}=\frac{1}{b-n}\overset{n}{\underset{a=1}{\sum }}\frac{|\left|V_{p}{\left(f_{r}\right)}_{D}-V_{p}{\left(f_{r}\right)}_{0}\right||}{V_{p}{\left(f_{r}\right)}_{0}}$$

where:

${\left|V_{p}\left(f_{r}\right)\right|}_{0}$: absolute value of the voltage output signal as extracted from the PZT at the healthy pristine state of the specimen,
${\left|V_{p}\left(f_{r}\right)\right|}_{D}$: absolute value of the corresponding voltage output signal as measured from the same PZT at damage level D,
b,n: number of date points in the moving frame,
M: number of measurements in the frequency band 10–250 kHz.

The pluralism of the applied indices demands an increased workload for collecting and editing the inserted data correctly and an increased workload for observing and analyzing the results among the different indices. However, employing different indices for the statistical analysis of damage detection could be a further step to verging a complete damage quantification methodology.

### 3.3. Damage Evaluation of the T-FRP beam

The schematic presentation of the codified names and positions for all the T-FRP beam’s PZT transducers is displayed in Figure 3b.

**Figure 8:**
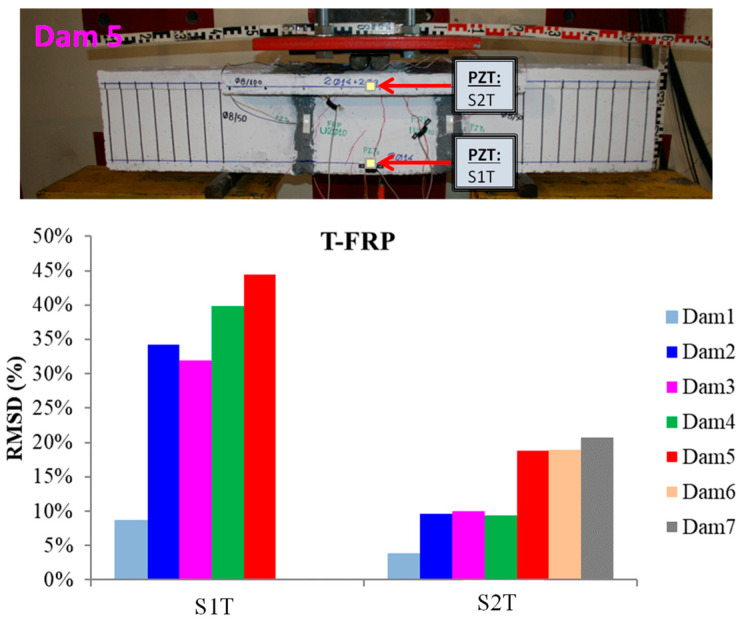
RMSD index values of PTZs mounted on the steel reinforcing bar of T-FRP.

The RMSD volume ratios of the epoxy-bonded PZT patches to the tensional and compressional reinforcement steel bars TS and CS, respectively, are depicted in Figure 8. The values show that a net of stresses was developed near the monitoring area of transducer S1T from the early loading stages, which turned out to be from the fact that the first flexural crack had already been formed in the 2nd WiAMS’ measurement (Dam2). Furthermore, due to the propagation of the flexural crack, which vertically intersected the position of the PZT transducer S1T, a considerable alteration in the RMSD index value was observed at the second damage state.

The increment of loading, the development of tensional stresses in the middle zone of the rebar TS, and the effect of the transported stresses by the concrete over the interface of the piezoelectric transducer led to ascending RMSD values up to the fifth damage state measurements, where the yielding point of the rebar occurred. This was the last acquired EMI method measurement, as the functionality of the transducer was possibly affected by the intense concentration of tensional stresses subjected to the interfacial with the concrete region, or its welded wires had been destroyed probably by the extensive cracking formation near the area of the PZT.

The rebar TS failed at the seventh damage level, where the reinforcement bar was cut into two separate pieces, unable to withstand the developed deformation, as shown in Figure 8. The failure and the high concentration of tensional stresses were probably caused due to the notch grooving over the rebar operating as a socket of the internal epoxy-bonded PZT transducer S1T.

Additionally, the internally epoxy-bonded PZT transducer S2T also showed an ascending pattern of RMSD values from the early loading states, which kept stable up to the fourth damage state where an increment of the RMSD ratio volume appeared, reaching a 20% volume ratio. The lower performing values of the RMSD index for PZT transducer S2T compared to the corresponding transducer S1T are due to the significantly lower developed stresses subjected to steel reinforcement bar CS, as it was placed in the compression zone of the T-shaped beam where the development of stresses is limited.

**Figure 9:**
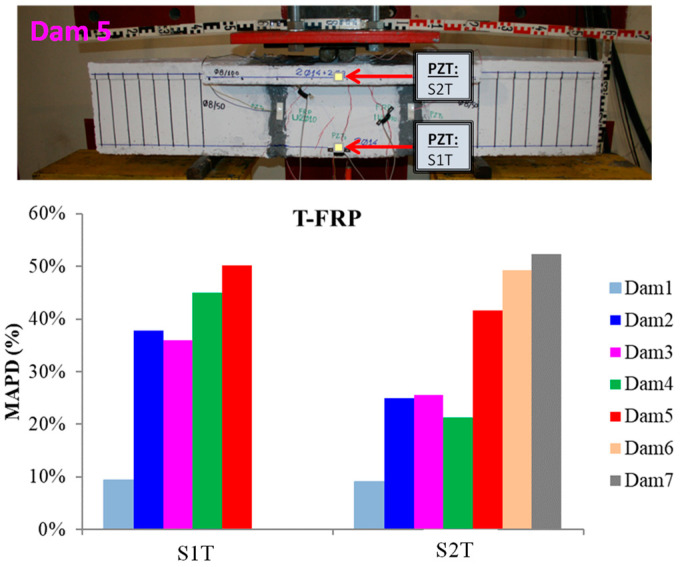
MAPD index values of PTZs mounted on the steel reinforcing bar of T-FRP.

Furthermore, in Figure 9, the pattern of changes among the bars of damage index MAPD are presented. Concerning the results of PZT transducer S1, the volume ratios are approximately equal to the relative of the RMSD index, while the relevant ones of PZT transducer S2T show almost doubly increased volume ratios compared with the corresponding RMSD damage states. Therefore, it could be assumed that the extracted MAPD damage index seems more sensitive to changes in the electro-mechanical signatures showing higher volume ratios.

An important observation is that the damage index volume ratio increased when the cracking propagated along the height of the beam until it reached the joint with the slab intersecting vertically with the position of the PZT transducer S2T.

**Figure 10:**
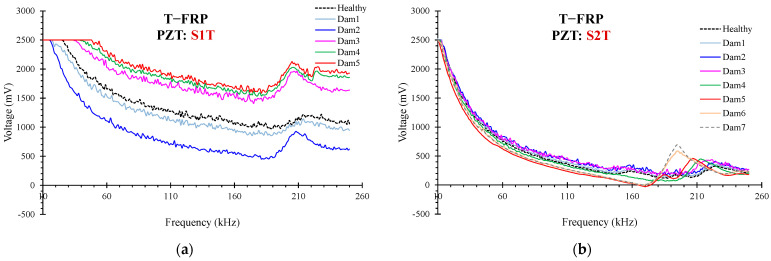
Typical curves of the electro-mechanical signatures of PZT transducers (**a**) S1T and (**b**) S2T.

Typical curves of the electro-mechanical signatures of PZT transducers S1T and S2T are illustrated in Figure 10. From the presented figure, it could be extracted that there is a slightly decreased shifting of the peak voltage response frequency concerning the damage progression for PZT transducer S1T. Furthermore, for PZT transducer S2T, there is a homologous downshifting of the peak voltage response frequency but in a swifter ratio as shown in Table 2.

Eventually, the changes caused in the specimen through the progressive loading process led to modifications to the position and the shape of the acquired voltage responses compared to the pristine condition. Consequently, it could be assumed that the peak frequency response alterations measured at the end of each loading step depict the material’s damage state in the transducer’s contiguity [53].

**Figure 11:**
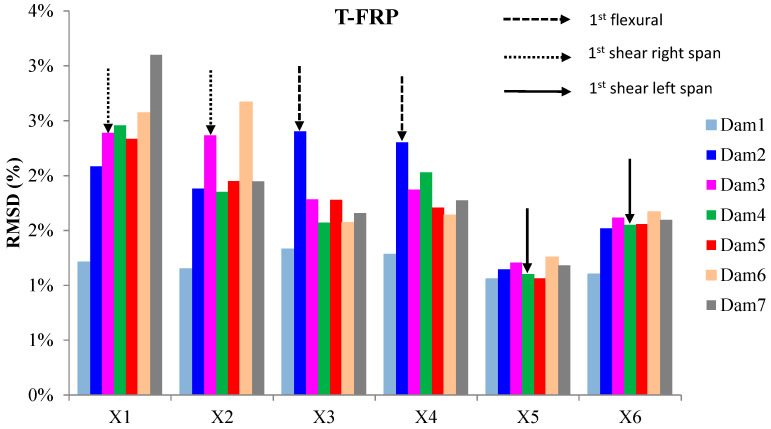
RMSD index values of PTZs externally epoxy-bonded on the surface of T-FRP.

**Figure 12:**
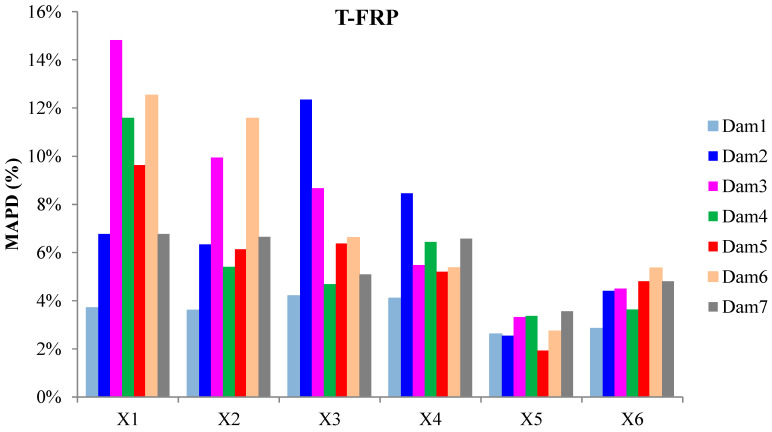
MAPD index values of PTZs externally epoxy-bonded on the surface of T-FRP.

The externally bonded PZT patches showed notable performance at certain damage states, even though their values of damage indices are significantly presented with a lower magnitude order (Figure 11 and Figure 12). Each piezoelectric patch, regarding its mounted position, exhibited a particular efficacy concerning the substrated formed crack and potential damage. The proximity of the PZT transducers to the corresponding cracks affected the performance of the damage index at each particular damage state.

Furthermore, the damage indices’ values of PZTs X1, X2, X5, and X6 were not solely affected by the formed cracks but also by the activation or deactivation of the C-FRP ropes. In detail, PZTs X3 and X4, which were mounted in the bottom of the mid-span, as shown in Figure 3b, showed a sudden increment in both damage indices analysis (RMSD and MAPD) at the second damage state when the first flexural crack was formed, while PZTs X1 and X2 showed the same behavior at the third damage state when the first shear crack appeared to the right span.

On the other hand, PZTs X5 and X6 did not fully exhibit the pre-described trend at the fourth damage state, as the first left shear crack was not formed widely and was so close to the PZT patches’ position.

**Figure 13:**
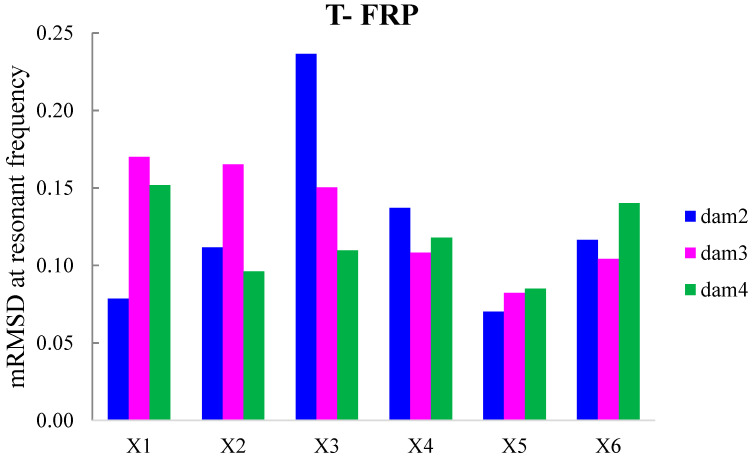
mRMSD at the resonant frequency of PTZs externally bonded on the surface of T-FRP.

**Figure 14:**
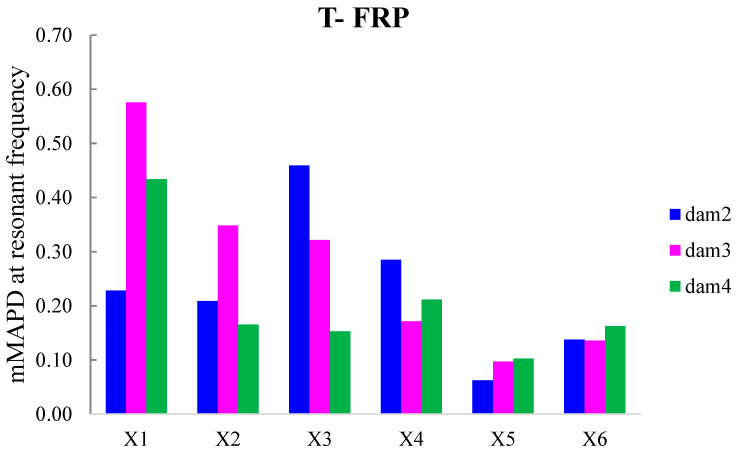
mMAPD at the resonant frequency of PTZs externally bonded on the surface of T-FRP.

In addition, the dynamic metrics of moving RMSD (mRMSD) and moving MAPD (mMAPD) were employed at a frame of overlapped moving blocks of 50 consecutive data points to enhance the damage location process. Furthermore, the moving indices were implemented for a frequency range of 100–150 kHz within which the resonant frequency of the external-bonded PZTs was included. Figure 13 and Figure 14 depict the moving RMSD and the moving MAPD charts at the resonant frequency for each externally bonded PZT patches for the damage cases 2, 3, and 4, where the first flexural, the first right shear, and the first left shear cracks were formed, respectively.

### 3.4. Damage Evaluation of the R-FRP beam

**Figure 15:**
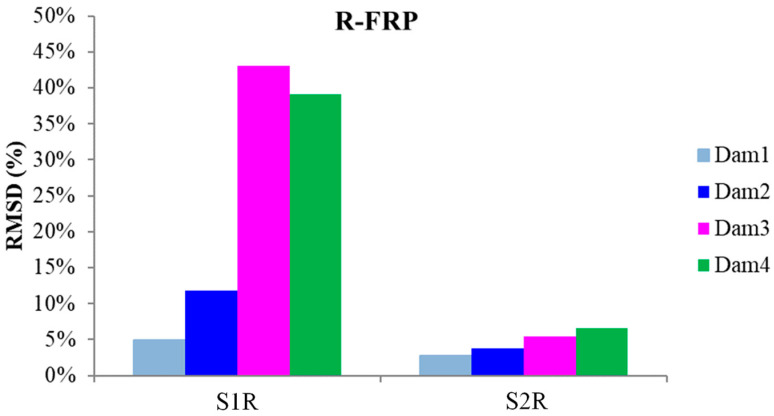
RMSD index values of PTZs mounted on the steel reinforcing bar of R-FRP.

The schematic presentation of the codified names and positions for all the R-FRP beam’s PZT transducers is displayed in Figure 3a. The RMSD volume ratio of the epoxy-bonded PZT patches to the tensional and compressional reinforcement steel bars TSR and CSR, respectively, are depicted in Figure 15. The values show similar behavior with the corresponding T-FRP ones and those stresses were developed near the monitoring area of PZT transducer S1R from the early loading stages, which turned out from the fact that the first flexural crack had already been formed in the 1st WiAMS’ measurement as shown in Figure 6 (Dam1). Furthermore, due to the propagation of the flexural crack, which vertically intersected the position of the PZT transducer S1R, significant changes in the RMSD index value were observed at all the subsequent damage states.

The progressive increment of the subjected load developing tensional stresses in the middle section of the rebar TSR and the impact of the developed stresses over the interface of the piezoelectric transducer by the concrete led to increasing RMSD values up to the fourth damage state, where the yielding point of the rebar occurred. That was the last occupied WiAMS measurement, as the functionality of the transducer was possibly affected by the intense concentration of tensional stresses subjected to the interfacial with the concrete region, or its welded wires had been destroyed probably by the extensive cracking formation near the area of the PZT. Finally, the rebar TSR failed after the fifth damage level, where it was cut into two separate pieces, unable to withstand the developed deformation, as shown in Figure 6. The failure and the high concentration of tensional stresses were probably caused due to the notch grooving over the rebar operating as a socket of the internal epoxy-bonded PZT transducer S1R.

Reinforcement bar TSR similarly failed to the corresponding TST of the T-FRP beam. The epoxy steel-bonded PZT transducer S1R also showed similar performance with almost identical values of RMSD damage index to the corresponding T-FRP steel-bonded transducer S1T.

Additionally, the internally epoxy-bonded PZT transducer S2R showed a constant pattern of RMSD values up to the fourth damage state, where an increment of the RMSD value appeared, reaching a 10% volume ratio. The lower performed values of the RMSD index for PZT transducer S2R compared to the corresponding S1R is due to the significantly lower developed stresses subjected to steel reinforcement bar CSR, as it was positioned in the compression zone of the beam where the development of stresses is limited.

**Figure 16:**
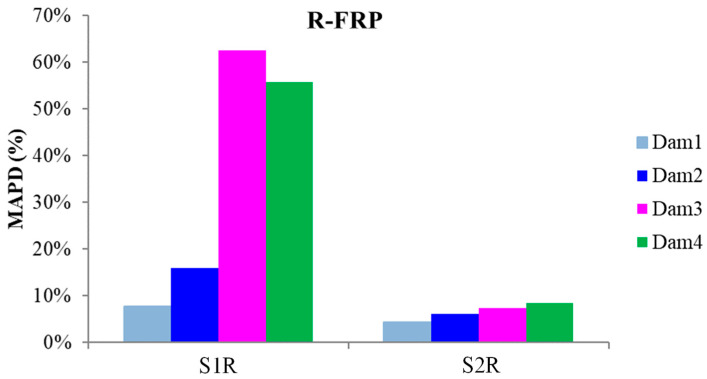
MAPD index values of PTZs mounted on the steel reinforcing bar of R-FRP.

Furthermore, Figure 16 presents the pattern of changes among the states of damage index MAPD. Concerning PZT transducer S1R, the volume ratios are higher at the third and fourth damage state compared to the corresponding RMSD index, even though both indices follow the same trend. In addition, the values of transducer S2R are almost similar to the corresponding RMSD ones and are also closely relevant to those extracted by the S2T transducer of beam T-FRP at the proportional damage level.

**Figure 17:**
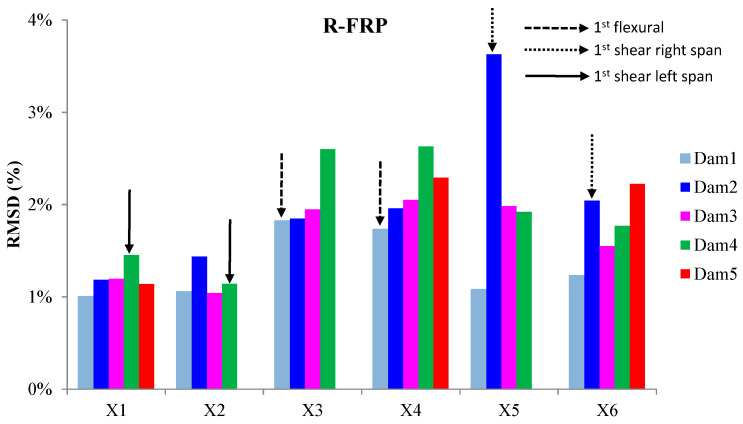
RMSD index values of PTZs externally epoxy-bonded on the surface of R-FRP.

**Figure 18:**
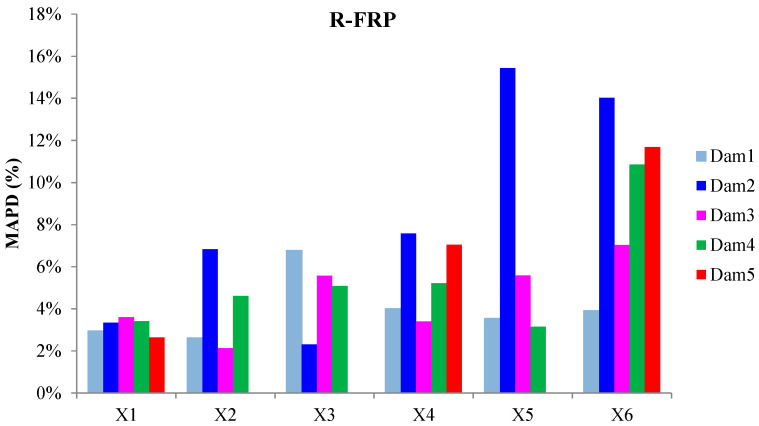
MAPD index values of PTZs externally epoxy-bonded on the surface of R-FRP.

The externally bonded PZT patches showed similar performance to the relevant of T-FRP beam and notable values at certain damage states. Each patch, regarding its mounted position, exhibited a particular efficacy concerning the substrated formed crack. The proximity of the PZTs to the corresponding cracks affected the performance of the damage index at each particular damage state. In detail, PZTs X3 and X4, which were mounted in the bottom of the mid-span, as shown in Figure 3a, showed a sudden increment in both damage indices analysis (RMSD and MAPD) at the first damage state when the first flexural crack was formed, while PZTs X5 and X6 showed the same behavior at the second damage state when the first shear crack appeared to the right span (Figure 17 and Figure 18). On the other hand, PZTs X1 and X2 did not fully exhibit the pre-described trend, as the left shear cracks were not formed widely and so close to the PZT patches’ positions as this was met to the PZTs X5 and X6 of the T-FRP beam.

**Figure 19:**
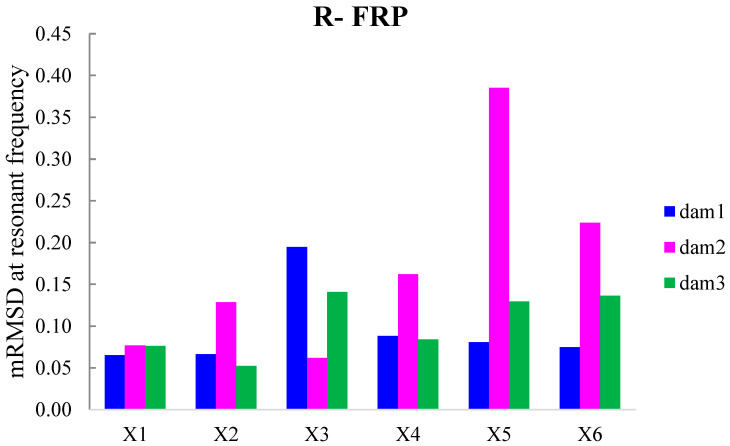
mRMSD at the resonant frequency of PTZs externally bonded on the surface of F-FRP.

**Figure 20:**
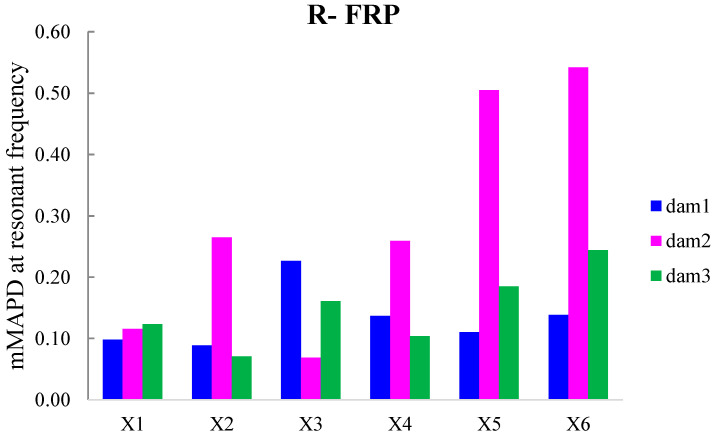
mMAPD at the resonant frequency of PTZs externally bonded on the surface of F-FRP.

Additionally, the dynamic metrics of moving RMSD (mRMSD) and moving MAPD (mMAPD) were implemented at a range of overlapped moving blocks of 50 consecutive data points to enhance the damage location process. Furthermore, the moving indices were applied for a frequency window of 100–150 kHz within which the resonant frequency of the external-bonded PZTs was inset. Figure 19 and Figure 20 depict the moving RMSD and the moving MAPD charts at the resonant frequency for each externally bonded PZT patch for the first, second, and third damage cases where the first flexural, the first right shear, and the first left shear cracks were formed, respectively.

**Figure 21:**
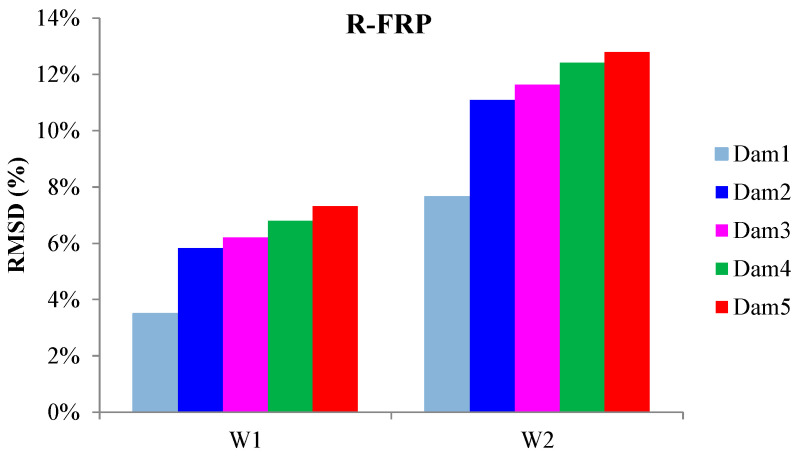
RMSD index values of PTZs mounted on the C-FRP rope of R-FRP.

**Figure 22:**
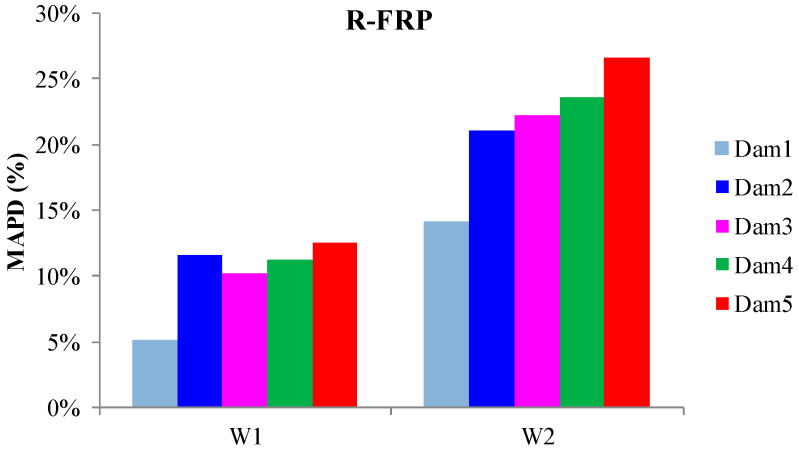
MAPD index values of PTZs mounted on the C-FRP rope of R-FRP.

Moreover, Figure 21 and Figure 22 present the RMSD and MAPD values of PZTs W1 and W2, respectively. Both PZTs were embedded in the mass of the concrete beam in the middle of the drilled holes. The PZTs have been placed ensemble with the insertion process of the C-FRP rope to examine the ability of the EMI-based PZT-enabled SHM system to detect the changes to the retrofitted region. Specifically, the rope’s performance during the loading variations, the development of the shear cracking, the stress condition, and the identification of the debonding of the C-FRP rope.

As presented in Figure 21 and Figure 22 both PZTs showed results relative to the mechanical performance of each inserted rope electro-mechanical response. Regarding PZT W2, which monitored the diagonal inserted rope W2, the indices’ values follow precisely the mechanical response of the rope, which contributed as a shear resistance mechanism to restrain the shear failure. As a result, the more the C-FRP rope was subjected to tensional stresses, the more the indices’ values increased. Furthermore, PZT patch W1 showed the same trend, even in lower values, as the failure pattern near rope W1 was formed in more distributed cracks. Another parameter was that the rope’s direction was not vertically positioned to the shear diagonal cracking, so a proportionally lower load was transferred as an applied force to the C-FRP rope compared to the C-FRP rope W2.

### 3.5. Comparatively Cases

**Figure 23:**
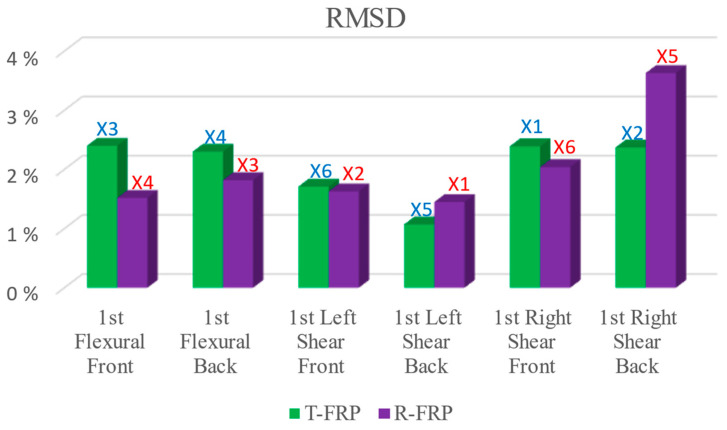
RMSD index values of externally bonded PTZs of T-FRP and R-FRP.

**Figure 24:**
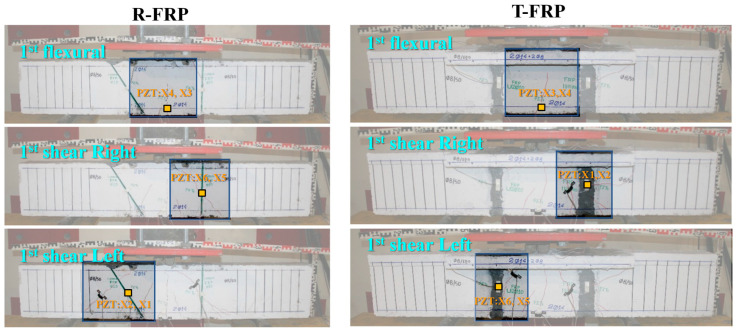
RMSD index values of externally bonded PTZs of T-FRP and R-FRP.

This sub-section presents comparative and synoptical diagrams of RMSD index values for T-FRP and R-FRP beams regarding the 1st flexural, right, and left shear span formed cracks. Figure 23 shows the RMSD values of the externally epoxy-bonded PZT patches for both beams calculated on the damage state where the 1st critical crack appeared in their monitoring area. Furthermore, Figure 24 depicts the areas of the beams where the critical cracks propagated and the positions of the externally epoxy-bonded PZT transducers. Combining the displays of the two figures, it could be assumed that for the 1st flexural crack, PZTs X3 and X4 of the T-FRP beam logically show higher values than those of beam R-FRP. This is because the flexural crack was formed right down the sensors and propagated at a lengthier distance at the height of the beam’s cross-section. Furthermore, PZT X5 of the R-FRP beam shows a higher value than the relevant PZT of beam T-FRP in the 1st right shear crack, connotating the rationale above. Finally, for the 1st left shear crack, PZT X6 of T-FRP shows a slightly higher RMSD index value conforming to the presented reasoning, which correlates the proximity of the PZT to the formed crack in its contiguity monitoring area with higher exhibited RMSD values.

In addition, a noteworthy point of the presented RMSD values of the externally bonded PZT patches is that the opposite-placed pairs (front and back) of the PZT transducers performed almost with the same sensitivity and could provide evidence of the fidelity of the proposed EMI-based SHM technique.

## 4. Conclusions

In this study, the efficiency of the EMI method for SHM of a retrofitting technique against shear for RC deep beams without steel stirrups using carbon fiber-reinforced polymer (C-FRP) ropes as transverse reinforcement was investigated. In addition, the EMI-based method was also implemented to investigate the ability of the PZT-enabled SHM system to detect the changes to the specimens’ health condition. Specifically, the proposed method was used to identify the debonding of the C-FRP rope, and to examine its efficacy in damage detection as far as the formed cracks’ location, distance, direction, and width.

Concerning the innovative shear strengthening technique, a summarized analysis of the experimental test results is gleaned from the conclusions below:

- Both shear strengthening techniques alter the performance of the tested beams to a ductile response. The proposed anchorage method of the fiber ropes enhanced their efficiency by increasing the bearing capacity and improving post-peak response in terms of strength and overall behavior. In addition, the fibers of the ropes did not exhibit rupture or debonding failure. Nevertheless, more experimental data are required to establish the efficacy of this technique.

From the EMI-based experimental results and the statistical analysis using different damage indices, it could be demonstrated that:

- The externally epoxy-bonded PZT transducers have successfully detected the loading/damage steps at which the first flexural crack and the initiation of the shear cracking occurred. Furthermore, SHM data support the effectiveness of the applied strengthening technique.
- The internal rope-bonded PZT transducers showed promising results, demonstrating a direct correlation with the mechanical performance of the C-FRP rope, thus contributing as an emerging tool for the early and real-time diagnosis of potential fiber debonding. However, the externally bonded PZTs showed less sensitivity in terms of damage indices’ values compared to the internally bonded ones.
- Cracking through the PZT’s position affects the transducer’s sensitivity as a significant change to the bonding parameters between the patch, and the host structure was observed. The impact of this condition could not be yet quantified numerically, but it should be considered in the evaluation of the acquired data as a probable condition of false damage alert.
- The reliability and sensitivity of the proposed PZT-enabled monitoring technique has been indicated since PZTs placed on the steel reinforcement at the same positions in all of the testing beams (S1T/S1R and S2T/S2R) demonstrate a similar trend of damage indices at the corresponding damage levels. Nevertheless, more tests are required to establish this method for the localization of the cracks and prompt damage detection.

## Figures and Tables

**Table 1:** Mechanical properties of strengthened materials.

Material	Mechanical Properties	
Impregnated C-FRP rope SikaWrap FX-50C	Laminate tensile strength	2100 GPa
	Laminate modulus of elasticity in tension	230 GPa
	Laminate elongation at break in tension	0.87%
Sikadur 300	Tensile strength	45 MPa
	Modulus of elasticity in tension	3.5 GPa
Sikadur 330	Tensile strength	30 MPa
	Modulus of elasticity in tension	4.5 GPa
Sika Anchorfix 3+	Sikadur 3+ compressive strength	114 MPa

**Table 2:** Peak voltage response frequency.

Condition	Frequency (kHz)	
	S1T	S2T
Healthy	228	225
Dam 1	214	225
Dam 2	208	224
Dam 3	209	221
Dam 4	206	213
Dam 5	205	207
Dam 6	-	196
Dam 7	-	195

## Data Availability

The data presented in this study are available on request from the corresponding author.
